# Co^2+^ Substituted Spinel MgCuZn Ferrimagnetic Oxide: A Highly Versatile Electromagnetic Material via a Facile Molten Salt Route

**DOI:** 10.3390/nano10122333

**Published:** 2020-11-25

**Authors:** Lankeshwar M. Thorat, Digambar Y. Nadargi, Mohaseen S. Tamboli, Abdullah M. Al-Enizi, Rahul C. Kambale, Shoyebmohamad F. Shaikh, Shard S. Suryavanshi, Mohd Ubaidullah, Ayman Nafady, Mohammed A. Al-Abdrabalnabia

**Affiliations:** 1 School of Physical Sciences, Punyashlok Ahilyadevi Holkar Solapur University, Solapur 413255, India; lankeshwarthorat@gmail.com; 2 Department of Chemistry, Hanyang University, Seongdong-gu, Seoul 04763, Korea; tamboli.mohseen@gmail.com; 3 Department of Physics, Savitribai Phule Pune University, Pune 411007, India; rckambale@gmail.com; 4 Department of Chemistry, College of Science, King Saud University, Riyadh 11451, Saudi Arabia; sshaikh1@ksu.edu.sa (S.F.S.); mtayyab@ksu.edu.sa (M.U.); ayma@ksu.edu.sa (A.N.); moh_chemical@hotmail.com (M.A.A.-A.)

**Keywords:** MgCoCuZn ferrites, molten salt route, magnetic properties, initial permeability, electric properties

## Abstract

We report on the electromagnetic properties of Co^2+^ substituted spinel MgCuZn ferrites developed via a facile molten salt synthesis (MSS) route. The choice of synthesis route in combination with cobalt substitution led to strong electromagnetic properties such as high saturation magnetization (i.e., 63 emu/g), high coercivity (17.86 gauss), and high initial permeability (2730), which are beneficial for the multilayer chip inductor (MLCI) application. In a typical process, the planned ferrites were synthesized at 800 °C using sodium chloride as a growth inhibitor, with dense morphology and irregularity in the monolithicity of the grains. The compositional analysis of as-prepared ferrite confirms the presence of desired elements with their proportion. The crystallite size (using X-ray diffraction (XRD) analysis) for different samples varies in the range of 49–51 nm. The scanning electron microscopy (SEM) and transmission electron microscopy (TEM) analysis showcases the compact morphology of the developed samples, which is typical in the ferrite system. The dielectric properties (dielectric-loss and dielectric-constant) in the frequency range of 100Hz–1MHz suggest normal dielectric distribution according to interfacial polarization from Maxwell–Wagner. From the developed ferrites, upon comparison with a low dielectric loss with high permeability value, Mg-Cu-Zn ferrite with Co = 0.05 substitution proved to be a stronger material for MLCIs with high-performance applications.

## 1. Introduction

**Figure 1:**
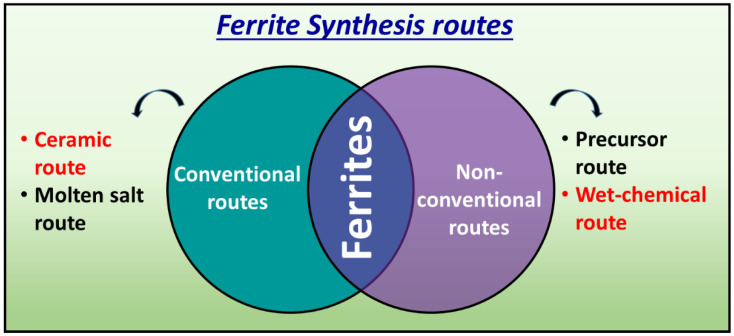
Various routes of ferrite synthesis.

Spinel ferrites are one of the most important subjects for magnetism and associated uses in various kinds of electromagnetic system [1,2,3]. The magnetic properties in these materials derive from the interactions between metallic ions the oxygen ions. Therefore, it comes to a special stand where the preparation strategies play an important role as illustrated in Figure 1. In the state-of-the-art, various synthetic protocols are being developed to materialize the versatile ferrites for diverse applications. The comparative chart of most exploited synthesis routes along with their merits and demerits are tabulated in Table 1 [4].

Maite Insausti and co-workers reported the analytical chemistry route to develop the ferrite nanoparticles for magnetic hyperthermia treatments. The synthesis strategy has helped to achieve larger-sized particles, which have the highest defined rate and thus are most suitable for applications of magnet hyperthermia [5]. Hui Xia et al. developed the multifunctional hetero-architectural copper ferrite/graphene composite via a hydrothermal route for photocatalysis and energy storage applications. The adopted route has provided a high surface area needed for the aforementioned applications [6]. Klaus Becker and coworkers disseminated the results of nickel ferrite, fabricated using the mechanochemical synthesis method. The work shows that the magnetic characteristics of NiFe_2_O_4_ particles can be adjusted by properly regulating their size [7]. Likewise, M. Bououdina, et al. showcased the fabrication of the varieties of spinel ferrites using a microwave combustion method for discrimination and elimination of lead and cadmium ions [8]. Furthermore, research has been conducted through hydrothermal pathways in relation to the ferromagnetic resonance properties of Mg/Zn nanoparticles, by the group of Chien [9]. To address the constricted yield after synthesis, various complementary techniques are available, such as molten salt, sol-gel auto-combustion, and the pyrolysis route [10,11,12]. The molten salt route is a conventional and well-developed synthetic method among the numerous synthesis techniques. It is known for being one of the easiest, most flexible and inexpensive ways of obtaining a high degree of quality ferrite in a shorter reaction cycle relative to the standard solid-state reactions. By considering its adequacies, we have developed the nickel-substituted MgCuZn ferrite using the molten salt route and investigated their electromagnetic properties useful for multilayer chip inductor (MLCI) application along with their structural and morphological analyses [13]. Meticulously, Mg-Cu-Zn ferrites are economically viable and need comparatively low sintering temperatures (<1000 °C).

Herein, the conventional pathway is, therefore, readily extended for fabricating cobalt-substituted MgCuZn ferrite using a molten salt route and its ferrite-related properties are investigated. The prime motivation for integrating Co^2+^ is due to its quick relaxing characteristic, which helps to improve the magnetic response time and microwave properties. Furthermore, Co^2+^ has a higher magnetic moment (3.87 μ_B_) compared to Mg^2+^ (1.1 μ_B_). Therefore, low eddy current losses, high electrical resistivity, high μ_i_, and Ms can be achieved in the pristine ferrite [13]. The synthesized material has been characterized by means of XRD, SEM, TEM, electrical and magnetic (VSM) analysis in order to get about the phase, morphology, electrical resistivity and magnetic behavior of the material. The procedures used in the characterizations process have been detailed in supporting (SI) information.

## 2. Experimental Details

**Figure 2:**
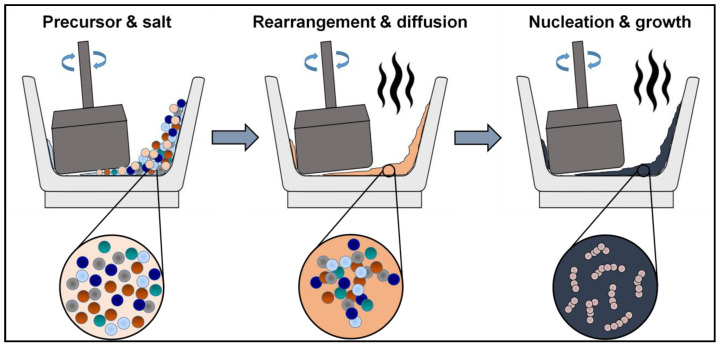
Schematic of ferrite development via molten salt route.

The divalent metal precursors (Mg, Co, Cu, Zn) were taken in the form of sulfates, whereas Fe source was taken as nitrate (Fe(NO_3_)_3_⋅9H_2_O). The reaction was carried out in the presence of NaOH and NaCl. The molar ratio of synthetic reagents was taken as: sulphates: Fe-nitrate: NaOH:NaCl:1:2:8:10. The mixture of the reaction was ground in an agate mortar for 1 h. The reaction began voluntarily with the heat release. The mixture turned mushy and eventually changed from colorless to black, with the exothermic reaction progressing. The schematic of the synthesis is highlighted in Figure 2.

The mixture obtained was collected in a crucible and sintered at 800 °C for 2 h. To overcome the characteristic property of NaCl as a particle growth suppressor, the chosen sintering temperature corresponded to NaCl melting point (801 °C). Subsequently, the material was allowed to come down to ambient atmospheric conditions, and rinsed thoroughly with deionized water, followed by drying under the infrared (IR) lamp. This powder was made into pellets as well as toroids, and sintered at 900 °C (4 h) in air, to obtain proper phase formation of the desired ferrite. The characterizations of the developed material can be found in our earlier reports, published elsewhere [13].

## 3. Results and Discussion

### 3.1. Crystallographic Identification

**Figure 3:**
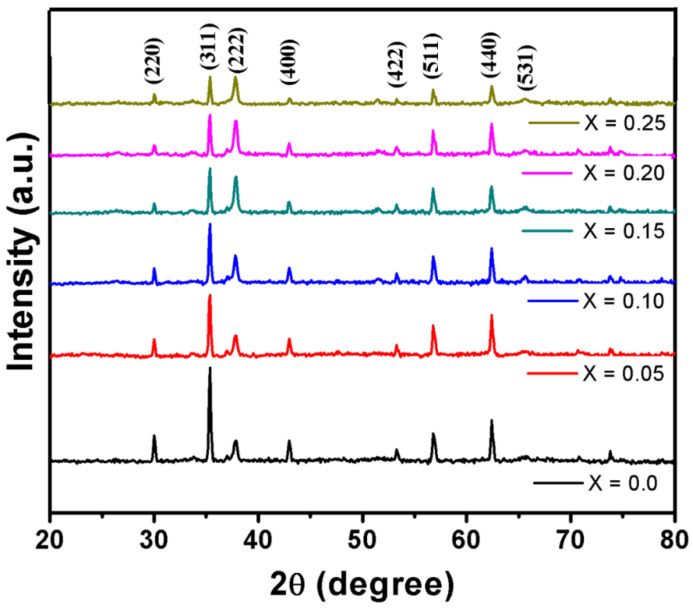
X-ray diffraction (XRD) graphs of the sintered Mg_0.25−x_Ni_x_Cu_0.25_Zn_0.5_Fe_2_O_4_ ferrites.

The X-ray diffraction (XRD) spectra in Figure 3 show the Bragg reflections of Co^2+^ substituted MgCuZn ferrite, a typical cubic spinel structure. The peaks (220), (311), (222), (400), (422), (511), (440) and (531) correspond to the standard data with JCPDS files no. 08-0234. Fine particulates of developed polycrystalline ferrite can be visualized with the wide and well-resolved peaks in the XRD patterns. The lattice constant (Table 2) was found to be influenced by the addition of Co with invariant behavior and is comparable with earlier reported values [14,15]. The range of average crystallite size was calculated to 48–52 nm, with no particular dependency on the amount of Co added. This confirms the adopted synthesis technique is favorable to develop the nanosized Mg-Cu-Zn ferrite powders with ease. As tabulated in Table 2, the samples displayed a relatively high density of nearly >90% which was due to the substitution of Cu ions in the ferrite system. Owing to its high mobility in terms of atomic scale, its presence facilitates the densification of the ferrite, which enhances the diffusion of cations in the lattice [16,17]. It can also be noticed from Table 2 that the density values of the samples, employing X-rays, are higher than the bulk measurements. This is due to the presence of pores in the bulk measurements [18].

### 3.2. Morphological and Elemental Analyses

**Figure 4:**
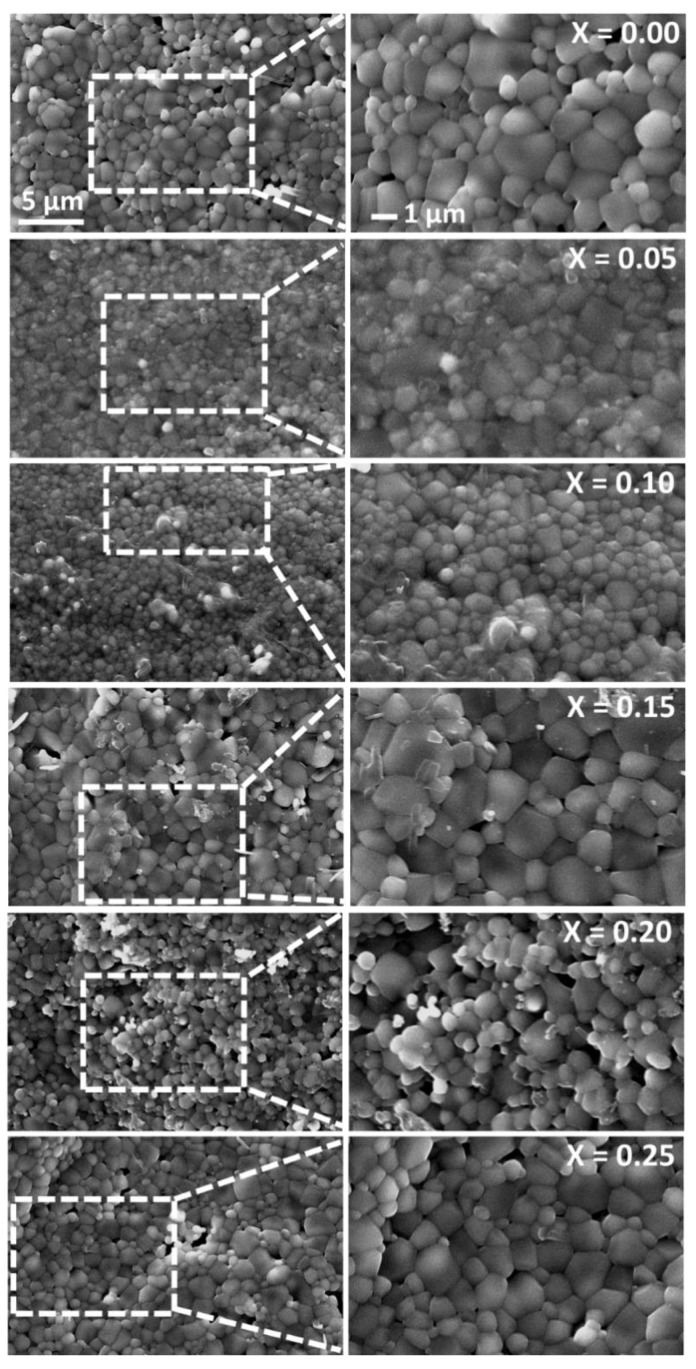
Morphological analysis of sintered Mg_0.25−x_Co_x_Cu_0.25_Zn_0.5_Fe_2_O_4_ ferrites.

**Figure 5:**
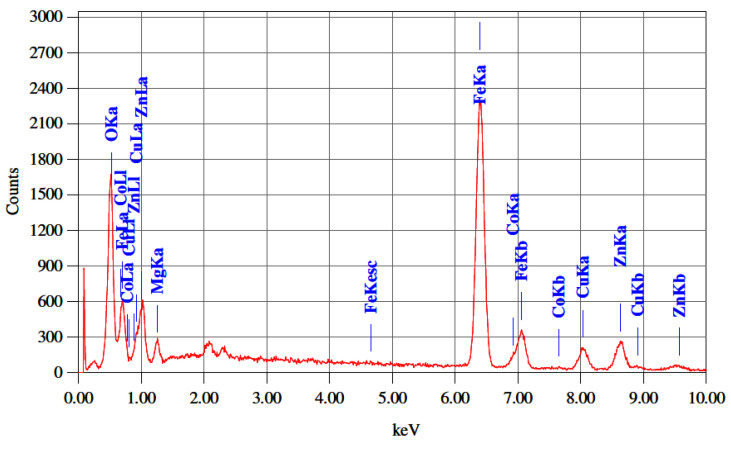
EDX analysis of sintered Mg_0.25−x_Co_x_Cu_0.25_Zn_0.5_Fe_2_O_4_ ferrite sample at x = 0.05.

The microstructure of the ferrite has a profound influence on its electrical and magnetic properties. Figure 4 shows the micrographs of all the developed ferrites from x = 0 to 0.25. The effect of Co^2+^ substitution has no specific trend in grain growth. Nonetheless, a uniform grain structure can be seen in the individual ferrite. Cu plays a key role in achieving a particular microstructure because of the creation of a liquid phase. This makes the grain growth much easier due to its separation at the grain boundaries. The reason behind the segregation is the increased rate of cation inter diffusion due to Cu^2+^ ion [19]. The thermal energy creates a force during the sintering process that causes the grain borders to expand over pores, thereby reducing the pores’ volume and making the product dense. The presence of Co substitution was confirmed using energy-dispersive X-ray spectroscopy (EDX) analysis (Figure 5, Table 3). The relative substitution of Co in Mg_0.25−x_Co_x_Cu_0.25_Zn_0.5_Fe_2_O_4_ ferrite was found to be optimal.

**Figure 6:**
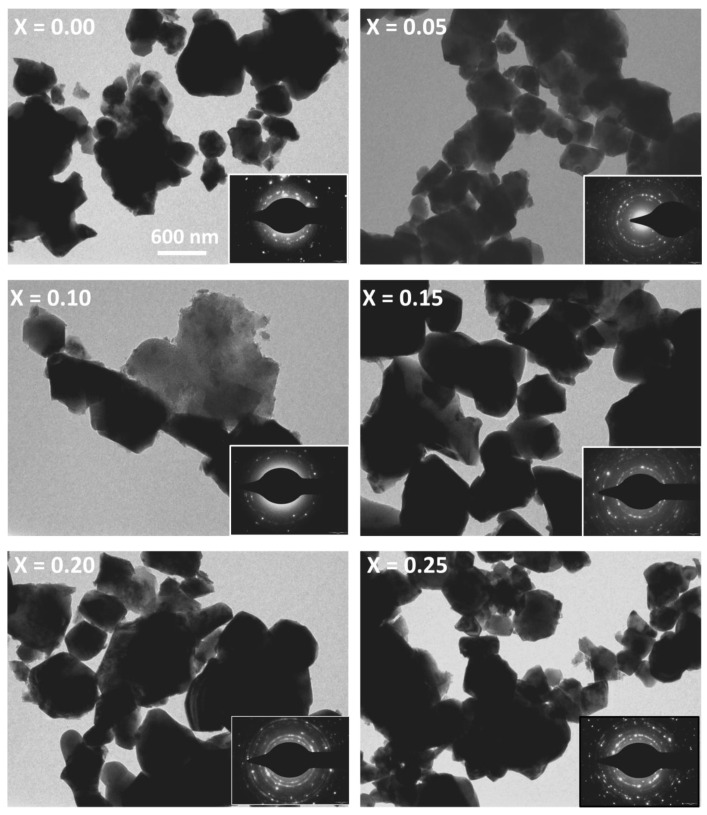
Micrographs (TEM and SAED) of sintered Mg_0.25−x_Co_x_Cu_0.25_Zn_0.5_Fe_2_O_4_ ferrites.

Figure 6 shows the typical TEM images of the developed ferrites. The TEM images show well-dispersed aggregates of particles. However, it is quite difficult to predict the particular shape of the particle, however, the average particle size for various compositions was found to be in the range of 118–314 nm (see Table 4). The particle size was found to be influenced by the addition of Co and the observed maximum for x = 0.25. Here as well, it is revalidated that there is no certain trend of increase/decrease in the particle size with increasing Co addition in the native ferrite structure. The TEM images (Philips CM 200 FEG) showed a kind of agglomeration, that may occur from the interface tension resulting from the magnetic surfaces or the exchange between the magnetic particle interactions. Also, some degree of agglomeration during sintering is unavoidable [20]. The selected area electron diffraction (SAED) pattern of the developed ferrites shows the presence of bright spot rings corresponding to various crystal planes of polycrystalline ferrite as seen in the XRD analysis.

### 3.3. Electrical Properties

#### 3.3.1. Direct Current (DC) Resistivity Studies

**Figure 7:**
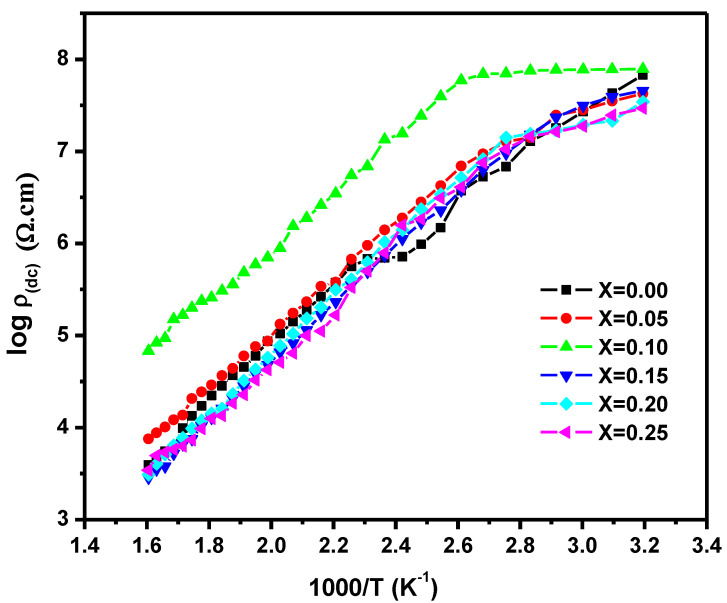
The log function of direct current (DC) electrical resistivity with respect to reciprocal of temperature for sintered Mg_0.25−x_Co_x_Cu_0.25_Zn_0.5_Fe_2_O_4_ ferrites.

Electrical DC resistivity is a major aspect of low-temperature sintered materials for high-frequency MLCI applications. The ferrite resistivity was known to depend on its composition and sintering temperature. The temperature shift of the DC electrical resistivity from room temperature to 350 °C for the developed ferrites is shown in Figure 7. The plot shows that with a rise in temperature, resistivity for all samples was reduced. Furthermore, the plot revealed the existence of many regions with various slopes due to changes in the mechanism of conduction. The mechanism of conductivity at low temperatures is due to impurities, while it is due to the alteration of magnetic order above the Curie temperature. The room temperature resistivity increased with the Co addition and attained maximum value for a sample with x = 0.10. The resistivity decreased with the further addition of Co. The measured activation energies for various regions (Ferro, Para) and Curie are given in Table 4. It was verified that in the paramagnetic region, the observed activation energy values are higher than in a ferromagnetic region that corresponds to Irkhin and Turov’s theory [21].

#### 3.3.2. Dielectric Properties

**Figure 8:**
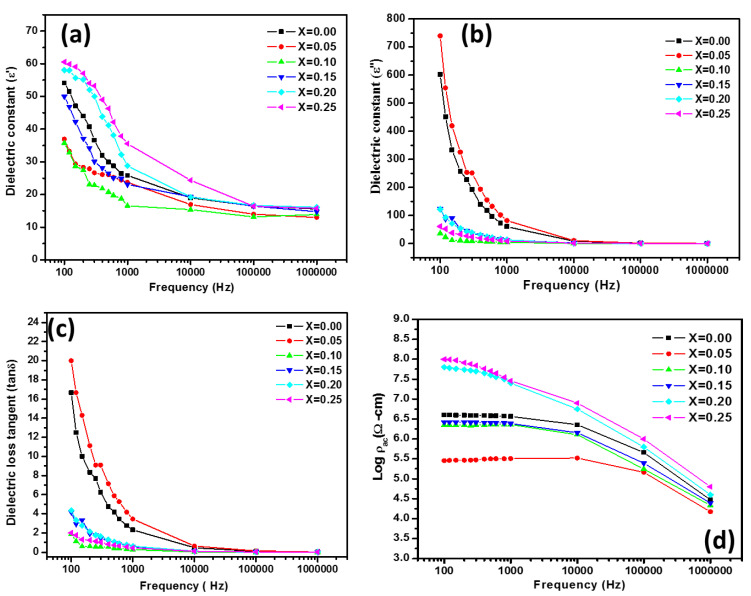
(**a**,**b**) Variation of dielectric constant, (**c**) dielectric loss tangent, and (**d**) alternating current (AC) resistivity with frequency for sintered Mg_0.25−x_Co_x_Cu_0.25_Zn_0.5_Fe_2_O_4_ ferrites.

Dielectric characteristics of ferrite provide important knowledge on the behavior of the localized electric carriers contributing to a better understanding of dielectric polarization mechanisms. The dielectric characteristics were analyzed in the frequency spectrum from 100 Hz to 1 MHz using an LCR meter (Hioki model 3532-50 LCR HiTester, Japan). Polarization of space charge and hopping process lead the dielectric reaction. Fe^3+^ and Fe^2+^ ions at octahedral B-sites contribute to dielectric polarization, with electric dipole rotation resulting in the polarization direction. When electrons are exchanged between the ions Fe^2+^ and Fe^3+^, and vice versa, interaction between the Fe^2+^ and Fe^3+^ takes place. The dipole then orients itself and the alignment slows. The sample material has a dielectric composition composed of two layers: first a ferrite grain layer, which is an extremely conducive medium, divided from an affected material layer of grain borders by a second thin layer. The dielectric constant (ε′) in all the ferrite specimens at 300 K is shown in Figure 8a. The initial frequency field, which remained constant in the higher frequency (over 100 kHz), showed a rapid decrease of the dielectric constant. For the sample with x = 0.10, the minimum dielectric constant was observed. All the samples exhibited the frequency dispersion behavior of dielectric in the lower frequency region. However, at a higher frequency, the dielectric remains unchanged. With the Co addition, the resistivity improved and the maximum value for a ferrite sample was reached with x = 0.10. It then declines with additional Co content.

The complex dielectric constant (ε″) is a physical quantity that shows the capacity of materials to cope with external fields which are highly dependent on frequency. For all sintered ferrite samples, Figure 8b indicates a dielectric constant (ε″) behavior. The dielectric constant decreases with an increase in frequency and at a higher frequency approach a constant value. All the samples show a frequency-independent behavior after a certain increase in frequency [22,23].

Figure 8c shows the frequency dependence in dielectric loss tangent. The dielectric loss tangent is reduced to a certain frequency with an increase in frequency. At higher frequencies, it remains almost constant. For high-frequency magnetic applications, low tan δ is required. On the role of Co substitution, the dielectric loss tangent got decreased with an addition of Co in Mg_0.25−x_Co_x_Cu_0.25_Zn_0.5_Fe_2_O_4_ ferrites except for sample with x = 0.05. The Mg-Cu-Zn ferrite with x = 0.05 possessed comparatively low dielectric loss with a high value of permeability which proves its candidature as a better material for MLCIs with high-performance applications.

The variation of alternating current (AC) resistivity for all the ferrite (Mg_0.25−x_Co_x_Cu_0.25_Zn_0.5_Fe_2_O_4_) samples are shown in Figure 8d. The ac resistivity in the cases decreased with an increase in the frequency. The ac resistivity at room temperature was found to vary with the addition of Co in Mg_0.25−x_Co_x_Cu_0.25_Zn_0.5_Fe_2_O_4_ ferrites. It increased with Co content and achieved a maximum for a sample with x = 0.25.

### 3.4. Magnetic Properties

The magnetic characteristics of soft ferrites are determined by the materials’ composition, morphology/microstructure, and the presence of additives in them. In the following, the magnetic properties are discussed in detail.

#### 3.4.1. Alternating Current (AC) Susceptibility

**Figure 9:**
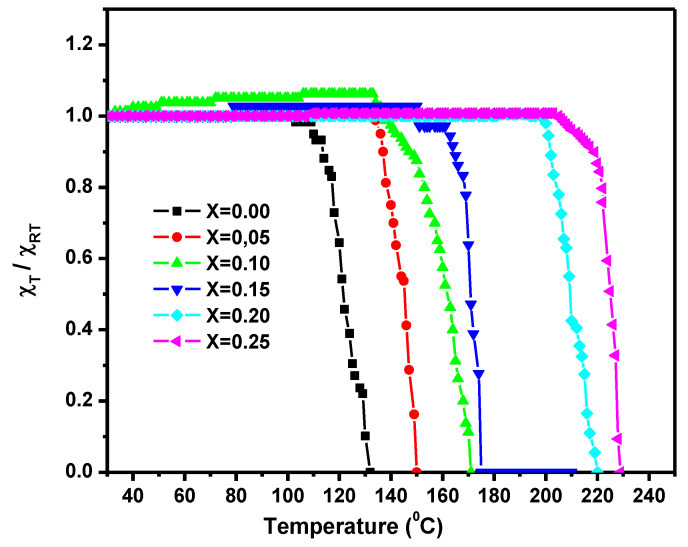
Normalized AC susceptibility (χT/χRT) associated to temperature for sintered Mg_0.25−x_Co_x_Cu_0.25_Zn_0.5_Fe_2_O_4_ ferrites.

The AC susceptibility with temperature for the sintered Mg_0.25−x_Co_x_Cu_0.25_Zn_0.5_Fe_2_O_4_ ferrites samples is shown in Figure 9. The normalized susceptibility of the developed ferrites found to be invariant to the temperature up to some definite limit and then exhibited a sudden decrease near the Curie temperature. The deep fall at Curie temperature indicated the formation of pure ferrite without any impurity [24]. The uniform AC susceptibility temperature invariance up to Curie temperature indicates that such compositions mainly involve multi-domain particles. The curves displayed a single peak behavior indicating that the substance does not contain impurity phases. The thermal energy was not sufficiently up to the Curie temperature to disrupt the synchronized spin moments. However, the thermal energy is high enough at the transition temperature to disrupt all the aligned spins, which are significantly less probable after the transition temperature. The obtained Curie temperatures from AC and DC susceptibility analyses match well. The Curie temperature increased as the cobalt content increased (Table 5). This is attributable to a rise in the ferrimagnetic region at the expense of the paramagnetic region, which contributes to an improvement of the contact between exchanges. If the magnetic interaction is stronger, the resultant Curie temperature will be larger. Furthermore, with the substitution of a high magnetic moment element (i.e., Co), the susceptibility at room temperature was found to increase with Co content.

#### 3.4.2. Magnetization

The magnetic hysteresis in the case of all samples exhibited a typical shape and confirmed that the samples are magnetically ordered. The maximum value of saturation magnetization was exhibited by x = 0.25 ferrite samples. The enhancement in saturation magnetization is achieved by highly magnetic Co^2+^ in place of non-magnetic Mg^2+^ ions.

**Figure 10:**
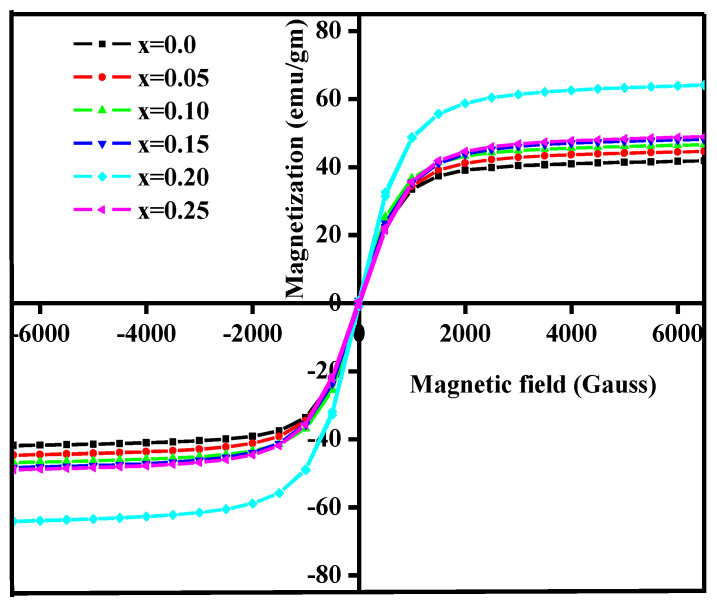
Magnetic hysteresis loops of sintered Mg_0.25−x_Co_x_Cu_0.25_Zn_0.5_Fe_2_O_4_ ferrites.

Figure 10 shows magnetic hysteresis curves of different samples of sintered Mg_0.25−x_Co_x_Cu_0.25_Zn_0.5_Fe_2_O_4_ferrites at room temperature. All the samples exhibited a typical magnetic hysteresis loop, indicating that the samples are magnetically ordered. The nature of the hysteresis loops indicated the soft ferromagnetic nature in all the samples. The values of saturation magnetization are given in Table 5. The saturation magnetization increased with the incorporation of Co with a maximum value of magnetization for a sample with x = 0.20.

#### 3.4.3. Initial Permeability

**Figure 11:**
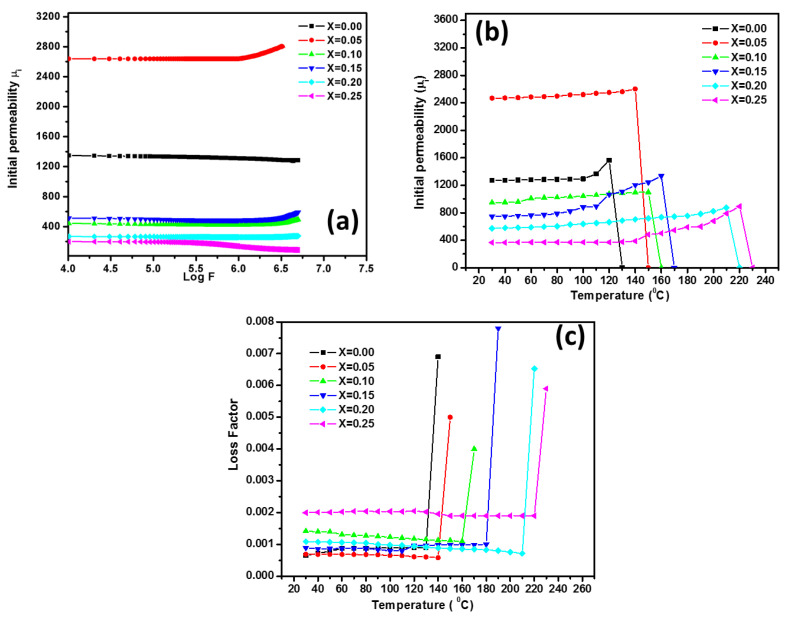
(**a**,**b**) initial permeability as a function of frequency and temperature, respectively, (**c**) loss factor as a function of temperature, of all the developed Mg_0.25−x_Co_x_Cu_0.25_Zn_0.5_Fe_2_O_4_ ferrites.

The initial permeability (μ_i_) was measured for all the ferrite samples in the frequency range of 100 Hz–5 MHz (Figure 11a). The initial permeability of all samples was almost steady up to a certain frequency and, thereafter, started to increase. The room temperature values of initial permeability (μ_i_) at 1 kHz are given in Table 6. An increase in the initial permeability may primarily have originated from the substitution of Co^2+^ ions for non-magnetic Mg^2+^ ions in the magnetic ferrite lattice. The high initial permeability for the sample with x = 0.05 is mainly due to the high relative density and grain size possessed by that sample (x = 0.05).

Figure 11b shows the initial permeability variation associated to. temperature of all the developed ferrites. As a function of temperature, the permeability value increased up to Curie temperature and reached a peak value, which thereafter dropped abruptly to a minimum value. An initial increment in permeability with the temperature is attributed to a quick reduction in the anisotropy field, instead of a decrement in the saturation moment [25]. The temperature at which a magnetic transition from ferrimagnetic to paramagnetic state occurs is the Curie transition temperature. As mentioned earlier, Mg_0.25−x_Co_x_Cu_0.25_Zn_0.5_Fe_2_O_4_ ferrite with x = 0.05 exhibited maximum initial permeability and, with a further increase in the Co amount, the permeability decreased. The Curie transition temperatures for all samples are given in Table 6. The degree of Curie transition temperature increases with the substitution of non-magnetic Mg^2+^ ion with Co^2+^ ions [26]. This is normal because cobalt is a magnetic substance and thus the gradual substitution of Mg^2+^ ions by Co^2+^ raises the temperature of the Curie transition. Since the initial permeability is directly proportional to saturation magnetization, the higher initial permeability value at x = 0.05 could be due to higher sintered density, higher grain size and positive magnetocrystalline.

From Table 6, it is seen that the wall permeability μ_w_ is very higher than rotational permeability μ_rot_ for all compositions of Mg_0.25−x_Co_x_Cu_0.25_Zn_0.5_Fe_2_O_4_ ferrite. Thus, it is concluded that the main contribution to the initial permeability is due to domain wall motion and hence the Globus model is applicable. Table 7 tabulated the heating and cooling cycle values of the initial permeability. All the ferrite samples demonstrated initial permeability thermal hysteresis and ∆μi improved with rising Co content.

The sample loss factor stays nearly stable at a certain temperature and increases afterward (Figure 11c). The higher initial permeability values are observed in the present case of samples. This is because the domain wall motion contributes more, as the sintered density and grain dimensions of the ferrite increase. The regulation of both the density and the grain size, which depends on the sintering conditions, will generally achieve higher initial permeability [27].

## 4. Conclusions

In conclusion, for the synthesis of Co-substituted MgCuZn ferrite, we have established a simple path, i.e., molten salt route. The Co substitution level in the parent ferrite was studied in between 0 and 0.25 (step 0.05), with Mg_0.25−x_Co_x_Cu_0.25_Zn_0.5_Fe_2_O_4_ being a system. The adopted molten salt route resulted in the cubic spinel structure of the ferrite system, as confirmed by XRD analysis. The lattice constant was found to be influenced by the addition of Co with invariant behavior. The uniform grain structure in the individual ferrites was shown by morphological analysis. Indeed, the creation of a liquid phase played a major role in the achievement of the specific microstructure. In an EDX study, the relative replacement for Co in ferrite Mg_0.25−x_Co_x_Cu_0.25_Zn_0.5_Fe_2_O_4_ was optimal. The SAED pattern of the ferrites reconfirmed the different crystalline planes of the developed ferrite. It was found that electric transport, dielectric and magnetic properties depend heavily on Co content. Dielectric and magnetic properties revealed that, given its high density, high initial permeability and saturation magnetization, Mg_0.25−x_Co_x_ Cu_0.25_ Zn_0.5_ Fe_2_O_4_ ferrite with x = 0.20 will serve as the possible candidacy for MLCI application.

## Figures and Tables

**Table 1:** Comparative chart of most exploited synthesis routes along with their merits and demerits.

#	Methods	Merits	Demerits
**1**	**Solvothermal**	Cost-effective	Long process time Need of autoclave for high pressure
**2**	**Hydrothermal**	Scalable, Cost-effective, High yield	
**3**	**Sonochemical**	Time effective, Fine size distribution	Less yield, uncontrolled particle shape
**4**	**Co-precipitation**	Facile synthesis route, almost water medium, good control on particle size and morphology, scalable	Broad size distribution of particle, weak crystallinity
**5**	**Thermal decomposition**	Great crystallinity, scalable, good control on particle size, narrow size distribution	Longer reaction time, high temperature, organic media, need of ligand exchange
**6**	**Sol-gel**	Facile synthesis route, great control on size, scalable	Alcoholic media
**7**	**Spray pyrolysis**	Variety of material deposition with ease	Control of nozzle, flow of liquid, high temperature
**8**	**Microwave assisted**	Very versatile, quick process time, narrow size distribution of particle	Low yield, need of microwave transparent reacting media
**9**	**Molten Salt**	Simple synthesis Low temperature	Low yield Longer time
**10**	**Ceramic**	Size control	Medium yield High temperature

**Table 2:** Properties of the developed ferrites on account of XRD measurements.

X	Lattice Constant A (Å)	Crystallite Size D (nm)	Bulk Density ρ_m_ (g/cm^3^)	X-Ray Density ρ_x_ (g/cm^3^)	Relative Density ρ_r_ (%)
0	8.411	48	4.62	5.14	90
0.05	8.406	48	4.97	5.18	96
0.10	8.417	49	4.88	5.20	94
0.15	8.394	52	4.96	5.28	94
0.20	8.411	49	4.86	5.29	92
0.25	8.414	49	4.94	5.32	93

**Table 3:** The EDAX statistics for different compositions of sintered Mg_0.25−x_Co_x_Cu_0.25_Zn_0.5_Fe_2_O_4_ ferrites.

Element	Mg_0.25−x_Co_x_Cu_0.25_Zn_0.5_Fe_2_O_4_ (Atomic %)					
	X = 0	X = 0.05	X = 0.10	X = 0.15	X = 0.20	X = 0.25
Mg	3.96	3.16	2.40	1.76	0.87	-
Co	-	0.77	1.61	2.48	3.10	4.27
Cu	4.14	4.17	4.39	4.29	4.50	4.36
Zn	8.37	8.18	8.29	7.83	8.27	8.63
Fe	31.97	32.67	32.10	33.19	34.83	34.99
O	51.56	51.05	51.21	50.45	48.43	47.75

**Table 4:** Avg. particle size from transmission electron microscopy (TEM), activation energy in Ferro and Para regions, and Curie temperature (T_C_) of sintered Mg_0.25−x_Co_x_Cu_0.25_Zn_0.5_Fe_2_O_4_ ferrites.

X	TEM-Avg. Particle Size (nm)	Activation Energy (eV)		Curie Temperature (°C)
		Ferro	Para	
0	314	0.35	0.55	131
0.05	118	0.39	0.60	160
0.10	226	0.36	0.60	167
0.15	255	0.39	0.64	192
0.20	195	0.41	0.68	210
0.25	299	0.24	0.51	232

**Table 5:** Details of magnetic properties of the developed ferrites.

X	M_s_ (emu/g)	M_r_/M_s_	n_B_Expt.	ρ_R_ (%)	H_c_ (G)	D (µm)	T_C_ (°C) χ_a.c._
0.00	42	0.0076	1.76	90	6.97	0.9	138
0.05	43	0.010	1.98	96	10.01	1.40	168
0.10	45	0.011	1.91	94	11.30	0.97	171
0.15	50	0.012	2.11	94	14.13	0.95	175
0.20	63	0.013	2.77	92	13.16	0.80	181
0.25	51	0.016	2.11	93	17.86	1.21	187

**Table 6:** Results of all permeability values along with magnetocrystalline anisotropy constant (K_1_).

X	Permeability			M_s_ (emu/g)	K_1_ × 10^4^ (erg/mL)
	Initial µ_i_	Rotational µ_rot_	Wall µ_w_		
0.00	1398	12	1257	42	−2.50
0.05	2730	30	2437	43	12.63
0.10	466	13	935	45	27.83
0.15	565	11	738	50	43.03
0.20	563	13	562	63	58.23
0.25	464	11	355	51	73.43

**Table 7:** Thermal hysteresis of initial permeability for developed ferrites.

X	µ_ih_	µ_ic._	∆µ_i_
0.00	1678	1689	11
0.05	2589	2601	12
0.10	1087	1099	12
0.15	1300	1334	34
0.20	834	892	58
0.25	800	890	90

## Supplementary Materials

The following are available online at https://www.mdpi.com/2079-4991/10/12/2333/s1, SI 1: Characterization of the material.
