# Supplementary material for: Co2+ Substituted Spinel MgCuZn Ferrimagnetic Oxide: A Highly Versatile Electromagnetic Material via a Facile Molten Salt Route
Source: Nanomaterials (Basel). 2020 Nov 25;10(12):2333. doi: 10.3390/nano10122333 (PMC7761114; doi:10.3390/nano10122333)
Supplement: Supplementary file 1 [file nanomaterials-10-02333-s001.pdf]

Supplementary information

Co^{2+} Substituted Spinel MgCuZn Ferrimagnetic Oxide: A Highly Versatile Electromagnetic Material via a Facile Molten Salt Route

Lankeshwar M. Thorat [†,1], Digambar Y. Nadargi [†,1,*], Mohaseen S. Tamboli [2,4], Abdullah M. Al-Enizi [3,*], Rahul C. Kambale [4], Shoyebmohamad F. Shaikh [3], Shard S. Suryavanshi [1,*], Mohd Ubaidullah [3], Ayman Nafady [3] and Mohammed A. Al-Abdrabalnabia [3]

[1] School of Physical Sciences, Punyashlok Ahilyadevi Holkar Solapur University, Solapur 413255, Maharashtra, India
[2] Department of Chemistry, Hanyang University, Seongdong-gu, Seoul-04763, South Korea
[3] Department of Chemistry, College of Science, King Saud University, Riyadh 11451, Saudi Arabia
[4] Department of Physics, Savitribai Phule Pune University, Pune-411007, Maharashtra, India
* Correspondence: digambar_nadargi@yahoo.co.in (D.Y.N.), amenizi@ksu.edu.sa (A.M.A.-E.) and sssuryavanshi@rediffmail.com (S.S.S.)
† These authors contributed equally.

SI 1. Characterization of the material

The ferrites were characterized by XRD, The XRD pattern, SAED; ED's analysis confirmed the spinel cubic structure and indicated the absence of any other impurity phase. The X-ray diffraction (XRD) pattern of the samples were obtained on BRUKER D8 advanced X-ray diffractometer using Cu-Kα (λ=1.54056 Å) radiation at 2θ values between 20º and 80°. The interplanar distance d (Å) was calculated using Bragg's law. The lattice parameter 'a' was calculated using the relation:

$$\frac{1}{d^2} = \left(\frac{h^2 + k^2 + l^2}{a^2}\right) \quad (1)$$

Where 'a' is the lattice constant, (h k l) are the Miller indices and 'd' is the interplanar distance. The average crystallite size (t) of the sintered powder was estimated using the Scherrer equation:

$$t = \frac{0.9\lambda}{\beta \, Cos\theta} \quad (2)$$

Where 'β' is the full width at half maximum (FWHM) for the most intense (311) reflection, 'θ' the angle of reflection, and 'λ' the wavelength of X-ray radiation used. The bulk density (ρ_m) was determined using the formula:

$$\rho_m = \frac{m}{\pi r^2 h} \quad (3)$$

Where m is the mass, r is the radius and h is the height/thickness of the pellet sample. The X-ray density (ρ_x) of the prepared samples was calculated from the relation:

$$\rho_x = \frac{ZM}{Na^3} \quad (4)$$

where 'Z' is the number of molecules per unit cell, which is 8 for the spinel cubic structure, 'M' is the molecular weight of the samples; 'N' is the Avogadro's number and 'a' is the lattice constant. Percentage porosity (P) of the samples was calculated from ρ_m and ρ_x values using the expression:

$$P(\%) = \left(1 - \frac{\rho_m}{\rho_x}\right) \times 100 \quad (5)$$

Scanning electron microscope (Model SEM-JEOL) operating at 5 kV was used to examine the surface morphology of the samples. Transmission electron microscope (TEM) images were taken by using a Philips CM 200 FEG microscope equipped with a field emission gun at an accelerating voltage of 200 kV, with a resolution of 0.24 nm.

The silver electrode paste was applied on two surfaces of the sintered pellet for dielectric, and electric property measurements. DC electrical resistivity measurements were carried out with two probe method using laboratory designed cell. We had measured the resistance of the sample as function of temperature. The pellet was mounted in a sample holder consisting of two silver rods in which the sample can be sandwiched with the help of screws. The cell was placed in a temperature controlled tube furnace. A calibrated chromel–alumel

thermocouple was used to measure the temperature of the tube furnace. The electrical resistivity was determined using the relation:

$$\rho_{dc} = \frac{\pi r^2 R}{t} \tag{6}$$

Where 'r' is radius, 't' is the thickness and 'R' is the resistance of the sample. Magnetization measurements of the powder samples were carried out using vibrating sample magnetometer (Lake Shore 7410 VSM). The magnetic hysteresis loops were measured with an applied magnetic field of 20 kOe. The susceptibility measurements were carried out on powder sample using double coil set up [39]. The toroid samples with outer diameter of 2.5 cm, inner diameter of 1.5 cm and thickness of 0.3 cm having 100 turns of winding were used for the inductance measurement. The inductance measurements were carried out from room temperature up to 350 ºC at 1 kHz using a computer controlled impedance analyzer (Hioki Model 3532-50 LCR HI Tester, Japan). Also the frequency dispersion of initial permeability was studied in frequency range 100 Hz to 5 MHz at room temperature. The initial permeability was determined from inductance measurements of coils using the formula:

$$\mu_i = \frac{L}{0.0046 N^2 h log_{10}\left(\frac{d_2}{d_1}\right)} \tag{7}$$

Where 'd_2' is the outer diameter, 'd_l' the inner diameter, 'L' the inductance in μH, 'h' the height in inches and μ_i the initial permeability. The dielectric measurements at room temperature were carried using LCR-Q meter (HP-4284A). The Curie temperature values (T_c) were determined from the temperature variations of susceptibility and initial permeability curves.
